# Innate Lymphoid Cells in Colorectal Cancers: A Double-Edged Sword

**DOI:** 10.3389/fimmu.2019.03080

**Published:** 2020-01-15

**Authors:** Qiutong Huang, Wang Cao, Lisa Anna Mielke, Cyril Seillet, Gabrielle T. Belz, Nicolas Jacquelot

**Affiliations:** ^1^Walter and Eliza Hall Institute of Medical Research, Parkville, VIC, Australia; ^2^Department of Medical Biology, University of Melbourne, Parkville, VIC, Australia; ^3^Olivia Newton-John Cancer Research Institute and La Trobe University School of Cancer Medicine, Heidelberg, VIC, Australia; ^4^The University of Queensland Diamantina Institute, The University of Queensland, Brisbane, QLD, Australia

**Keywords:** tumor immunology, adaptive immunity, innate immunity, tumor immunosurveillance, immunotherapy

## Abstract

The immune system plays a fundamental role at mucosal barriers in maintaining tissue homeostasis. This is particularly true for the gut where cells are flooded with microbial-derived signals and antigens, which constantly challenge the integrity of the intestinal barrier. Multiple immune cell populations equipped with both pro- and anti-inflammatory functions reside in the gut tissue and these cells tightly regulate intestinal health and functions. Dysregulation of this finely tuned system can progressively lead to autoimmune disease and inflammation-driven carcinogenesis. Over the last decade, the contribution of the adaptive immune system in controlling colorectal cancer has been studied in detail, but the role of the innate system, particularly innate lymphoid cells (ILCs), have been largely overlooked. By sensing their microenvironment, ILCs are essential in supporting gut epithelium repair and controling bacterial- and helminth-mediated intestinal infections, highlighting their important role in maintaining tissue integrity. Accumulating evidence also suggests that they may play an important role in carcinogenesis including intestinal cancers. In this review, we will explore the current knowledge about the pro- and anti-tumor functions of ILCs in colorectal cancer.

## Introduction

### Colorectal Cancer—Epidemiology, Pathophysiology, and Risk Factors

Colorectal cancer (CRC) is the third most common cancer worldwide with more than 1.8 million patients diagnosed each year (1). Despite intensified screening programs, most patients are diagnosed at the later stages of disease (2). This results in CRC emerging as the second leading cause of death with more than 900,000 deaths every year (1). CRC is a multifactorial and heterogeneous disease (3) with most cases being sporadic and fewer than 5% of patients with CRC have a positive familial history with inherited germline mutations that increase the risk of tumor development (3, 4).

Tumors are widely distributed from the proximal region of the colon to the rectum and each location is associated with different tumor-intrinsic characteristics (5). Most of these tumors arise from benign lesions called polyps, which progressively acquire genetic and epigenetic modifications to form malignant tumors and metastases (3, 4, 6, 7). These include deletions, amplifications, mutations or methylation of the promoter of certain genes such as *APC, SMAD4, TP53, BRAF, MYC, KRAS, PTEN*, or *MLH1* (3, 4, 8). The risk of developing CRC increases with age (4, 5) and is higher in men than in women (9). Other environmental factors such as exposure to smoking, alcoholic beverages, the presence of visceral fat and poor dietary patterns are all features associated with a higher risk of developing CRC (3, 5). In addition, inflammatory bowel disease (IBD) and chronic colitis have also been associated with an increased risk of CRC development (10, 11). However, improved anti-inflammatory treatments and increased surveillance have been effective in reducing CRC incidence for these patients (12).

### Treatment

The most effective first line treatment for patients diagnosed with localized CRC is surgery. In certain cases, neoadjuvant chemotherapy allows early reduction of the tumor burden to increase the chances of complete tumor resection (3, 4). For patients with metastatic CRC, there are increased treatment options available which include chemotherapies and targeted therapies.

#### Chemotherapy

Tumor recurrence occurs in 15–50% of patients who have undergone complete resection of loco-regional tumor lesions (4). To reduce this risk, patients often receive adjuvant chemotherapy treatment as a first line approach increasing both patient progression free and overall survival (13, 14). More recently, chemotherapy has been combined with targeted therapy to further improve patient overall response rates.

#### Targeted Therapies

Multiple treatment approaches have been developed in an endeavor to provide curative outcomes for patients. These include targeting oncogenic drivers (RAS, BRAF), receptors of growth factors (EGFR) or pathways involved in angiogenesis such as VEGFR (15). As an example, Bevacizumab, a human IgG1 antibody directed against VEGF-A, increased patient overall and progression-free survival when combined with chemotherapies (14, 16, 17).

Recent breakthroughs in tumor immunology have revolved around the clinical efficacy of therapeutic antibodies that are designed to block critical checkpoint molecules expressed at the membrane of circulating and tumor-infiltrating immune cells (18). Programmed death-1 (PD-1) blocking antibodies have been shown to induce remarkably durable clinical responses in many different cancer types, including CRC (19, 20). These anti-PD-1 sensitive CRC tumors harbor defective mismatch DNA repair mechanism (called microsatellite instability high or MSI high tumors) and is associated with enhanced mutational load, neoantigen formation, T cell infiltration of the tumor, and immune checkpoint expression (4, 8, 20, 21). However, only half of these CRC patients experience durable clinical responses (22) and many others develop resistance during treatment. To understand the underlying mechanisms, Grasso and colleagues performed large scale genomic analyses of more than 1,200 CRC tumors (23). This revealed that MSI high tumors have a higher rate of mutated genes in critical immune-modulating pathways such as antigen presentation that then drive resistance to treatment. In addition, upregulation of the WNT pathway in both MSI high and microsatellite stable CRC leads to a “cold” tumor microenvironment that is poorly infiltrated by anti-tumor T cells (23). Combined infusion of anti-CTLA-4 and anti-PD-1 blocking antibodies increased patient survival in metastatic CRC compared to anti-PD-1 treatment alone. However, it also induced higher levels of toxicity (24).

Currently, the use of immunotherapy and its integration into strategies for CRC care are growing (25). However, the quality of the immune response has not yet been fully factored into treatment decisions despite significantly influencing CRC patient outcomes. A better understanding of the tumor immune infiltrate would greatly inform treatment decisions and allow more strategic design of future combination therapies.

## The TUMOR Immune Contexture in Colorectal Cancer—the Success of the Adaptive Immune System

Most colorectal tumors develop from glandular epithelial cells of the colon or rectum (26) and evolve through close interaction with their microenvironment and diverse immune cell types. A critical balance between pro- and anti-tumor immune responses determines either the eradication of nascent lesions or the development and progression of transformed cells that form malignant tumors (27). It has been demonstrated that in patients where initial failure to eradicate emerging tumors occurs, immune cells still play a critical role in dictating the patient response to treatment and outcome (28–32). To account for the quality of the immune infiltrate, a scoring system called Immunoscore™ (33) has been developed by tracking adaptive immune cells such as CD3^+^CD8^+^ and memory (CD45RO^+^) T cells (Table 1). This allows the immune infiltrate and its location within the tumor sample to be integrated to stratify patients and accurately predict dissemination to distant metastasis (34), disease-free and overall survival (30, 31, 35) (Tables 1, 2). However, the impact of innate cells has not been considered. In this review, we will summarize the latest evidence implicating a role for innate lymphoid cells in CRC development and their impact on disease outcome.

**Table 1:** Parameters examined in Immunoscore™.

**Cell type**	Cytotoxic lymphocytes (CD3^+^CD8^+^) Memory T cells (CD45RO^+^)
**Location**	Tumor center Invasive margin
**Density**	0—low density of both cell populations in both regions 4—high density of both cell populations in both regions
**Prognosis-Cox analysis**	Disease free survival Overall survival Disease specific survival

**Table 2:** Immunoscore^TM^ and its association with the risk of relapse.

**Immunoscore**	**Risk of relapse**
0—low density of both cell populations in both regions	High
1—one cell population in one region	
2—one or both cell population(s) in one or both region(s)	
3—one or both cell population(s) in one or both region(s)	
4—high density of both cell populations in both regions	Low

## Role of the Innate Lymphoid Cells in Colorectal Cancer

**Figure 1:**
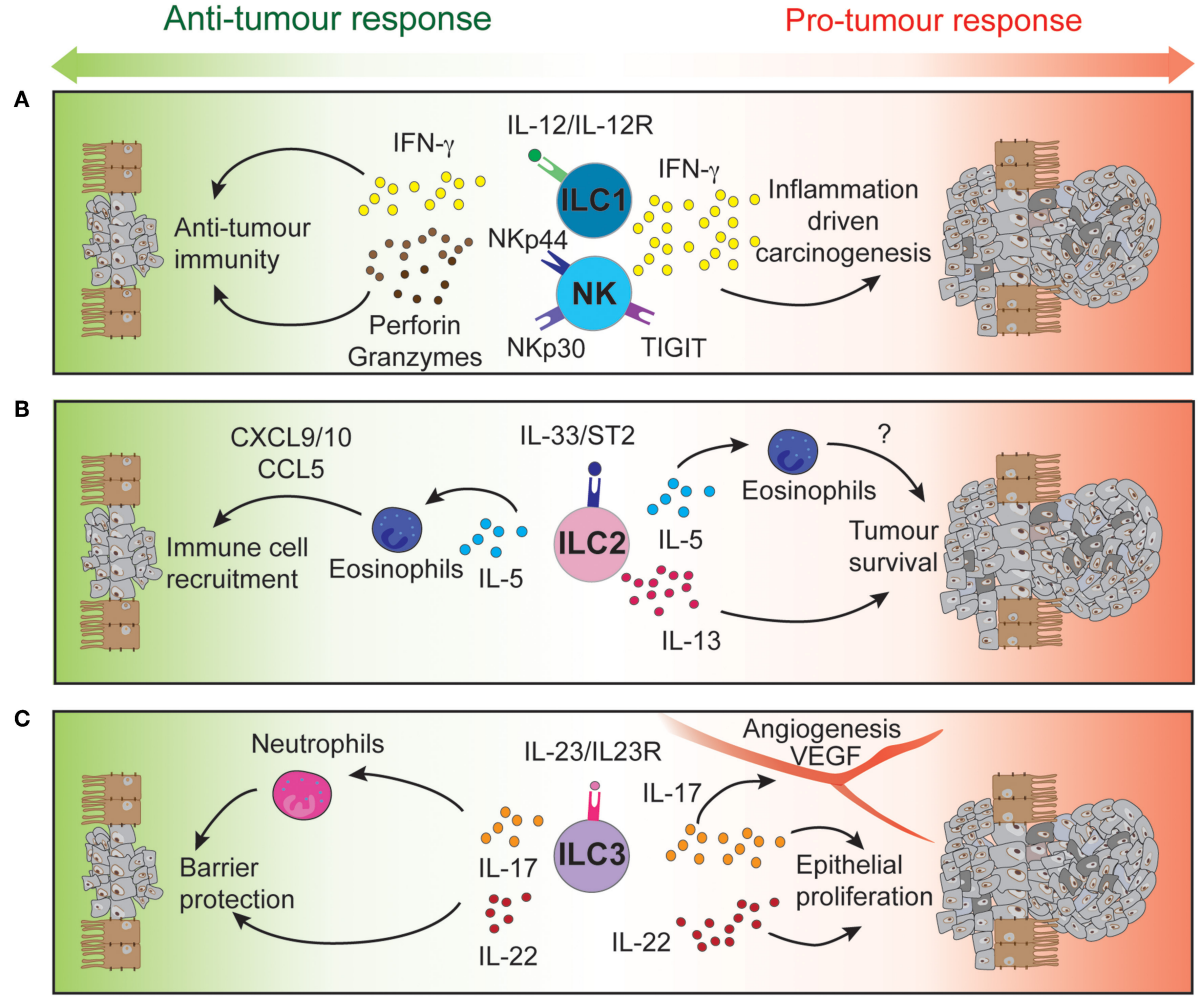
Intestinal ILCs drive both pro- and anti-tumor functions tipping the balance for tumor development. Signaling of NK cells and intraepithelial ILC1 **(A)**, ILC2 **(B)**, and ILC3 **(C)** through their activatory or inhibitory receptors regulates the function of these immune cells. Through the secretion of cytokines and cytotoxic molecules, ILCs can modulate the tumor microenvironment to either control or promote CRC development and progression.

Innate lymphoid cells (ILCs) are the innate counterpart of the T lymphocytes, mirroring key aspects of their phenotype and function. ILCs are divided into five subsets, classified based on their development, transcription factor expression, cytokine production and functions. These are NK cells, ILC1, ILC2, ILC3, and Lymphoid Tissue inducers (LTi) (36). They are enriched in mucosal tissues, including the intestinal tract (37) and respond to signals derived from their micro-environment such as cytokines, alarmins, and other inflammatory and non-inflammatory stimuli (38, 39) to drive appropriate immune responses and maintain tissue homeostasis. In addition, ILCs express particular receptors and ligands at their surface regulating further their function (40, 41). NK cells and ILC1 are mainly involved in the early protection against viruses (42), bacteria (43, 44), and cancer (45) through the secretion of interferon (IFN)-γ and granulocyte-macrophage colony-stimulating factor (46). ILC2 are essential in host protection against helminth and parasites throughout interleukin (IL)-4, IL-5, and IL-13 production (47). ILC3 express mainly IL-17 and IL-22, both cytokines critical to mucosal immune responses and epithelium regeneration (48). Finally, LTi are critical for lymphoid organogenesis (49). Beyond this, significant plasticity occurs between ILC subsets depending on environmental stimuli they receive, thus directly impacting on their effector functions and immune responses (50–55). While ILCs are essential to lymphoid organogenesis and tissue homeostasis, they also participate to the development of autoimmune diseases (56, 57) or inflammation-driven carcinogenesis (58) (Figure 1).

### Type 1 Innate Lymphoid Cells

High-dimensional cytometric analyses have revealed that human colorectal tumors are highly infiltrated by NK cells and intraepithelial-like ILC1 (59, 60). They secrete cytotoxic molecules such as granzymes and perforin (59, 60) and cytokines such as IFN-γ following stimulation (59), making them key potential players in cytotoxic anti-tumor responses (Figure 1A). In contrast, a separate study found impaired NK cell infiltration despite elevated levels of chemokines found in CRC tumors (61) (Table 3).

**Table 3:** Role of ILC in promoting or inhibiting CRC tumorigenesis.

**Immune cell type**	**Prognosis**		**References**
	**Anti-tumor**	**Pro-tumor**	
NK cells	• High cytotoxicity • Recognition of cancer initiating cells	• Low number of infiltrating NK cells • Reduced level of activating receptors (e.g., NKp44, NKp30) • Increased expression of inhibitory receptors (e.g., TIGIT) • Low cytotoxicity	(61, 64, 65, 67)
ILC1	• Produce IFN-γ and cytotoxic molecules associated with anti-tumor immunity^*^	• Accumulate in inflamed tissue • IFN-γ promotes inflammation^*^	(58, 62)
ILC2	• High pre-operative serum IL-13 • IL-5 recruits eosinophils	• Local IL-13 promote tumor epithelial survival and proliferation. • Elevated IL-5 in ulcerative colitis, anti-IL-5 reduced eosinophils and colitis^*^	(76, 77, 81–83)
ILC3	• IL-17 recruits neutrophils to protect tissue barrier^*^ • IL-22 promotes wound healing^*^ and protects intestinal epithelial cells from genotoxic stress-induced DNA damage	• Overproduction of IL-17 promote inflammation and angiogenesis, and disrupt the intestinal epithelial barrier • Overproduction of IL-22 dysregulates epithelial proliferation	(96, 102, 103, 106, 108, 109, 112, 113)

* *Indirect evidence of involvement in CRC development*.

Studies tracking intraepithelial ILC1s have shown that they accumulate in the intestine of patients with Crohn's Disease and in CRC (51, 59, 62). These intraepithelial ILC1s can be activated by chemokines and cytokines such as IL-18 produced by intestinal epithelial cells (63) or IL-12 from dendritic cells and monocytes (62). This induces IFN-γ production in ILC1 which, in contrast to previously mentioned studies (59, 60), drives the pathogenesis of anti-CD40-induced colitis in *Rag1*^−/−^ mice (62) (Table 3 and Figure 1A). Given these divergent outcomes, further studies are required to analyze the exact role of intraepithelial ILC1, their receptor expression and cytokine production using genetically-modified conditionally-deficient mice and human samples. How intraepithelial ILC1 influence patient outcomes and responses to therapy, is, to date, poorly understood.

NK cells are known to mediate anti-tumourigenic activity (45). In CRC, NK cells effectively eliminate CRC stem cells and cancer-initiating cells, which express lower levels of class I major histocompatibility complex (MHC) but high levels of ligands that bind the natural cytotoxicity activating receptors (NCR) NKp30 and NKp44. Consequently, these cells are “marked” for NK cell mediated lysis (64) providing a mechanism to potentially prevent tumor cell re-emergence or CRC metastasis. However, tumor cells can circumvent this process by differentiating and upregulating MHC class I expression to suppress NK cell activation or by reducing their expression of NCR ligands to escape NK cell-targeted killing (64). Thus, circulating NK cells in CRC patients are often phenotypically altered reflecting these induced changes. These patients exhibit reduced NKp44 expression on their CD56^dim^ cells (65), a key subset that normally exhibit potent effector function. In addition, high NKp30 expression on circulating CD56^bright^ cells has been associated with a shortened disease-free survival period in CRC patients (65). Other receptors and ligands expressed on NK cells of CRC patients such as activating and inhibitory KIRs (66) or TIGIT (67) have also been suggested to play distinct roles in the development, progression, and metastasis of CRC tumors (Table 3 and Figure 1A). In mice, neutralizing TIGIT markedly prolongs the survival of tumor bearing animals. However, when NK cells are depleted, this effect was completely abrogated highlighting the critical role of NK cells in this context. In addition, tumor infiltrating NK cells can transdifferentiate into ILC1-like cells in the presence of transforming growth factor-β (TGF-β) which is particularly abundant in the tumor microenvironment and associated with tumor progression (54). Does TGF-β accumulate in CRC tumors? If so, similar NK cell—ILC1 transdifferentiation could occur in CRC tumors and this would potentially with worsen prognosis. Collectively, it is clear that NK cells are involved in the control of CRC progression and metastasis but are also susceptible to tumor-directed dysregulation (41). To circumvent such events, current NK cell-based therapies are designed to boost NK cell functions in an effort to strategically further improve patient outcomes (68–71).

### Type 2 Innate Lymphoid Cells

The role of ILC2 has been examined in a number of different cancers including lung and gastric cancer (72, 73). Nevertheless, their role in colorectal cancer is, to date, only poorly understood. In fact, they are found to a relative lower frequency compared to other ILC subsets that reside in the intestine. Nevertheless, ILC2 frequency is increased in mucosal samples from IBD patients compared with healthy individuals (74). In CRC tumors, ILC2s express higher levels of ICOS and CD69 compared with ILC2 found in normal colon tissue (59, 60). These phenotypically distinct ILC2s could be a potential source of IL-4, IL-5, and IL-13 as both IL-4 and IL-13 are found at elevated levels in the serum of CRC bearing mice (75).

IL-13 can act directly on tumor epithelial cells in both an autocrine or paracrine manner to promote their survival and tumorigenesis (Figure 1B). In CRC, high expression of the IL-13Rα2 receptor is associated with more advanced disease stages, lymph node involvement, the presence of metastases and reduced survival (76). In contrast, Saigusa et al. found that low preoperative serum IL-13 was a predictive marker for poorer prognosis of colorectal cancer patients (77). These discrepancies indicate that IL-13 expression and its predictive value may be context dependent. The role of IL-5 has not been explicitly studied in the context of CRC. Nevertheless, it is important for eosinophil recruitment, expansion and activation (78, 79). In turn, these cells secrete chemo-attractants such as CXCL9, CXCL10, and CCL5 which promote the recruitment of CD8^+^ T cells to the tumor site (80) (Table 3 and Figure 1B). The presence of peritumoral eosinophils is an independent prognostic factor associated with favorable progression free survival (81). In contrast, patients suffering from ulcerative colitis, have a higher risk of CRC development and were found to have elevated IL-5 transcript levels (82). Neutralizing IL-5 in mice reduced eosinophil numbers and diminished the inflammation associated with DSS-induced colitis (83).

The role of ILC2s, their activation pathways and subsequent downstream signaling in CRC are highly contentious. This is because the same biomarkers or effector molecules (e.g., IL-5 and IL-13) have been found to exhibit opposing roles in CRC outcome, and thus, differentially impact patient prognosis. A similar effect is seen for the cytokine IL-33, a molecule released by damaged epithelial cells and regulator of ILC2 activation. Several studies have found both the cytokine IL-33 and its receptor ST2, to be elevated in mouse and human CRC tissues (84–86), suggesting a potential role of this IL-33/ST2 pathway in tumor development and progression. Mechanistically, IL-33 was found in adenomatous polyposis coli (*APC)*^*min*/+^ mice to promote intestinal polyp formation by activating stromal cells and modulating angiogenesis (85, 87), and by promoting cell growth and proliferation of primary CRC cells (88). Serum soluble ST2 levels were inversely correlated with the tumor stage despite being elevated in CRC patients. Indeed, soluble ST2 was shown to act as a decoy receptor suppressing IL-33-driven angiogenesis (89) and inhibiting CRC growth, while loss of ST2 expression conferred protection in AOM/DSS-induced CRC (90). Conversely, reduced levels of serum IL-33 is observed in CRC patients compared with healthy individuals and decreased expression of ST2L, the transmembrane isoform of the ST2 receptor, in CRC tumors is associated with higher tumor grade and worse prognosis (91). These conflicting results indicate that the IL-33/ST2 axis may playing a dual role in CRC pathogenesis and is capable of both promoting or protecting against CRC tumor development and progression depending on the environmental context. IL-33 has multiple downstream targets as ST2 is not only expressed on ILC2 but is also found on other intestinal cell types such as epithelial cells or regulatory CD4^+^ T cells (86). Therefore, the role of the IL-33/ST2/ILC2 pathway in CRC warrants further investigation using conditional mouse models, which would allow cell-specific responses to be disected.

### Type 3 Innate Lymphoid Cells

Inflammation is a key driver of cancer progression and the link between IBD and the development of CRC is well-established (92). This inflammation can be exacerbated by the loss of barrier protection occurring early during CRC development (93), allowing for the infiltration of microbes and their associated products into the tumor microenvironment. This activates dendritic cells and macrophages to produce IL-23 (94), a key cytokine regulating ILC3 function and plasticity (53, 95). Additional stimuli such as IL-12, IL-18, TGF-β, or Notch ligand can profoundly influence ILC3 plasticity (50–52). In the presence of IL-12 or TGF-β, IL-22-expressing ILC3 can acquire ILC1 features and transdifferentiate into IFN-γ producing cells (52). At the molecular level, the transcription factors T-BET, AIOLOS, and Signal Transducer and Activator of Transcription (STAT) 4 induce an ILC1 transcriptional program, repressing in parallel the ILC3 lineage genes (52, 53). Transitional ILC3/ILC1 cells were found in human intestinal epithelium (52) and accumulate in inflamed lesions of patients suffering from Crohn's disease (51) suggesting that such plasticity may also occur in inflamed CRC lesions.

IL-23 has been found to be overexpressed in both mouse and human CRC tumors (93, 96, 97) and IL-23 deficiency has been shown to protect against tumor development in a number of mouse models of cancer (97). Conversely, chronic IL-23 injection induces ILC3-dependent duodenal adenomas development in mice (98). Furthermore, local IL-23 expression drives ILC3 accumulation and activation within the colonic lamina propria (99, 100). These intestinal ILC3 express higher levels of the activating receptors ICOS and CD69 (59, 60), regulators of tumor responses CD244 and CD39 (59), and exhibit enhanced production of the downstream signaling cytokines IL-17 and IL-22 that contribute to the pathogenesis of CRC (96) (Table 3 and Figure 1C).

Several studies have reported high IL-17 expression in both mouse and human CRC compared with normal adjacent tissues (96, 101–103). ILC3s produce high levels of IL-17 but other immune and non-immune intestinal cell types also express this cytokine. These include Th17 cells, γδ T cells, and Paneth cells (104). Importantly, IL-17 transcripts are increased during progression of tumors from the adenoma to carcinoma stages in CRC patients (105). Furthermore, IL-17 can alleviate cell cycle inhibition, thereby promoting epithelial proliferation and intestinal inflammation (106) and increase angiogenesis by inducing VEGF expression (103). Administration of anti-IL-17 antibodies have been shown to ameliorate inflammation severity (96) while genetic ablation of IL-17 was able to reduce tumor burden (93, 100, 106, 107). Although, these studies show that IL-17 is a pro-inflammatory cytokine involved in CRC pathogenesis, IL-17 may also play a protective role. Lin and colleagues (102) reported better patient overall survival associated with high IL-17 levels (102) and neutralizing IL-17 exacerbated Crohn's disease and DSS-mediated colitis by increasing gut permeability (108, 109) (Table 1).

In a similar pattern to IL-17, the ILC3-driven IL-22 pathway plays both protective and pathogenic roles during colitis and tumorigenesis (Table 3). Under normal conditions, IL-22 is required for homeostatic epithelial cell proliferation through STAT3 activation (110, 111). Furthermore, IL-22 production by ILC3 and γδ T cells protects the intestine from genotoxic stress-induced DNA damage through effective initiation of the DNA damage response program (112). Loss of IL-22 or IL-22 receptor on epithelial cells results in delayed tissue repair, exacerbated inflammation (113) and tumor development when intestinal cells are exposed to carcinogens (112). Conversely, sustained IL-22-dependent epithelial proliferation leads to tumor progression (Figure 1C) and neutralizing IL-22 reduced CRC development (96). In addition to this, loss of IL-22 binding protein, a soluble decoy receptor neutralizing IL-22-driven cell activation, has been shown to increase epithelial cell proliferation and was associated with a higher tumor burden (114).

These studies unanimously suggest that IL-23 signaling in ILC3 is central in driving CRC development. Indeed, the absence of ILC3 despite chronic IL-23 injections protects mice from tumor development (98). However, the downstream cytokine IL-17 and IL-22 signaling pathways seem to play a dual role in CRC. These findings suggest that initial activation of the IL-23/ILC3 pathway is sufficient to trigger tumor development but additional mechanisms influence CRC progression and outcomes. Collectively, further investigations are warranted to shed light on the role of these cytokines in CRC pathogenesis.

## Concluding Remarks

Despite increasing evidence for the crucial role of the immune system in dictating patient's prognosis, the evaluation of the tumor immune cell infiltration in primary CRC is not yet fully integrated into assessments in routine clinical practice (115). It is, however, clear that adaptive immune cells, particularly CD8^+^ memory T cells, play an important role to limit tumor recurrence and prolong patient's survival (30). How adaptive immune cells are influenced by resident innate immune cell populations, the first line of immune defense, is still poorly understood (116). Direct and indirect evidence tend to suggest that ILCs could play a dual role in colorectal cancer. However, most of the aforementioned findings resulted from studying knockout mice or mice lacking adaptive immune cells. Thus, further studies are warranted to ascertain the role of ILCs in immunocompetent animals using genetically-modified conditionally-deficient mouse models. Collectively, given their function and strategic location, harnessing ILC responses would open up new possibilities through the development of combination therapies to further constrain CRC progression.

## Author Contributions

QH and NJ wrote the initial draft. WC designed the figure. All authors provided critical insight, edited, and approved the final version.

### Conflict of Interest

The authors declare that the research was conducted in the absence of any commercial or financial relationships that could be construed as a potential conflict of interest. The reviewer DW declared a past co-authorship with one of the authors GB to the handling editor.
